# Exposure factors of cadmium for residents in an abandoned metal mine area in Korea

**DOI:** 10.1007/s10653-016-9872-7

**Published:** 2016-09-26

**Authors:** Seung Chul Ahn, Jun Young Chang, Jung Sub Lee, Hwa Yon Yu, A-Ra Jung, Jee-Young Kim, Jong-Woo Choi, Young-Seoub Hong, Seung Do Yu, Kyounghee Choi

**Affiliations:** 1 Environmental Health Research Division, National Institute of Environmental Research, 42, Hwangyeong-ro, Seo-gu, Incheon, Republic of Korea; 2 Environmental Measurement and Analysis Center, National Institute of Environmental Research, 42, Hwangyeong-ro, Seo-gu, Incheon, Republic of Korea; 3 Department of Preventive Medicine, Dong-A University School of Medicine, 32, Daesingongwon-ro, Seo-gu, Busan, Republic of Korea

**Keywords:** Cadmium, Human exposure factor, Abandoned metal mine, Risk assessment, Rice grain

## Abstract

This study evaluated blood and urine cadmium (Cd) levels and human exposure factors for residents in an abandoned metal mine in Korea. We collected blood, urine, soil, water, and rice grain samples to analyze Cd concentrations and analyzed heavy metal concentration patterns in soil. We estimated the major exposure factor of Cd through non-carcinogenic risk assessment depending on exposure routes. The blood Cd concentration in the case group was 5.33 μg/L (geometric mean), significantly higher than that in the control group (1.63 μg/L, geometric mean). Urine Cd concentrations were also similar. The Cd concentrations in paddy soil (1.29 mg/kg) and rice grains (0.14 mg/kg) in the study area were higher than those in the control area (0.91 and 0.07 mg/kg, respectively). The analysis of heavy metal concentration in soil showed that the Cd levels in agricultural soil in the case group were attributable to the mine. The hazard quotient (HQ) of Cd by rice ingestion in the case group (1.25) was 2 times higher than that in the control group (0.6). We found that the HQ of rice ingestion contributed to more than 97 % of the total HQ, indicating that rice grains were the major exposure source. However, it is likely that the continuous intake of Cd-exposed crops led to chronic exposure among the residents in mine area.

## Introduction

Cadmium (Cd) is a natural element in the earth’s crust at a concentration of 0.1–0.5 ppm, the concentration may increase if there is a pollution source such as an abandoned metal mine, nearby. The half-life of Cd absorbed into a human body is 26 years or longer (ATSDR 2012; Sheikh and Smith 1980), and the biggest source of exposure to Cd in a general population is food, which accounts for about 90 % of the total intake (ATSDR 2012). Chronic Cd exposure is known to affect kidney function and bones (ATSDR 2012), and exposure of the general population to Cd must be reduced, as it ranks seventh in the Priority List of Hazardous Substance of the US Agency for Toxic Substances and Disease Registry (ATSDR 2015). Long-term exposure to Cd has been shown to affect the metabolic balance in the human body (Ling et al. 2016), and a previous study found an association between increased urine Cd and osteoporosis (Jung et al. 2012). The main factor of exposure to Cd through oral intake is rice grains; the Cd concentration in the general population in countries where the main staple food is rice, such as Korea and Japan, shows a trend of being higher than that in other areas (ATSDR 2012).

Apart from Cd-exposed food intake, the general population is exposed to Cd through environmental pollution in the area, such as that due to an industrial complex or an abandoned metal mine. The heavy metal pollution from waste ore generated during mining and outflow of mine water from abandoned mines, for which no mine reclamation and damage prevention projects have been implemented, causes environmental pollution that affects the health of residents in the vicinity (NIER 2012a; Kwon 2011). As of 2014, 2089 out of 2166 metal mines in Korea were abandoned owing to changes in the economic environment, such as worsening of the profit structure; these mines are managed through soil pollution surveys and mine reclamation and damage prevention projects (KMOE 2014; MIRECO 2014).

Among the 2089 abandoned metal mines located throughout Korea, 1268 (60 %) have caused mine hazards, namely soil contamination caused by waste rocks and mine tailing (MIRECO 2014). Among those, the number of mines for which mine reclamation and damage prevention projects have been enforced after 2007 is approximately 260 (MIRECO 2014); pollution sources, such as waste rocks and mine tailings, were left as they were in the remaining mines without applying any appropriate prevention measures.

Residents living near the abandoned metal mines were people aged ≥65 years who had lived in the area for 40 years or longer (Park et al. 2014). There is a high likelihood that residents have been exposed to the pollutants as they have resided in the areas for a long time after development and abandonment of the mines. Moreover, most of them have been engaged in agriculture and have used water from the mine or river for drinking or agriculture; because of this, the health of the residents may have been affected.

Health effect surveys for residents living near abandoned metal mine areas in Korea have been administered sporadically since the 1990s, when the potential health problems caused by environmental pollution were recognized (NIER 1996). The necessity of systematic management was highlighted by the health effect survey of residents in the area of the Samsan Jeil mine in 2004 (Kim et al. 2008).

In 2007, the Ministry of Environment in Korea (KMOE) proposed a long-term plan for systematically surveying on the health effects of heavy metals exposure through a plot survey on 358 abandoned metal mine areas that could cause soil contamination. The National Institute of Environmental Research in Korea (NIER) has been performing health effect surveys since 2008 in order of survey priority, and as a result of the Environmental and Health Effects Survey of Residents around 2nd Phase Abandoned Metal Mines in 2013, a JH abandoned metal mine area was discovered where the percentage of the residents whose blood Cd concentration was in excess of the World Health Organization (WHO) reference value (5 μg/L) (WHO 1996) was higher than that of other areas (NIER 2013a, b, c).

This cross-sectional study aimed to identify the major exposure factors and their contribution rates by re-evaluating the concentration of Cd in biological samples of residents in the JH abandoned metal mine area; in addition, Cd concentrations in environmental media, such as soil, water, and agricultural products, were investigated. We intended to evaluate the correlation between the concentrations of Cd in residents and the JH abandoned metal mines.

## Materials and methods

### Subjects of the study and survey period

**Fig. 1:**
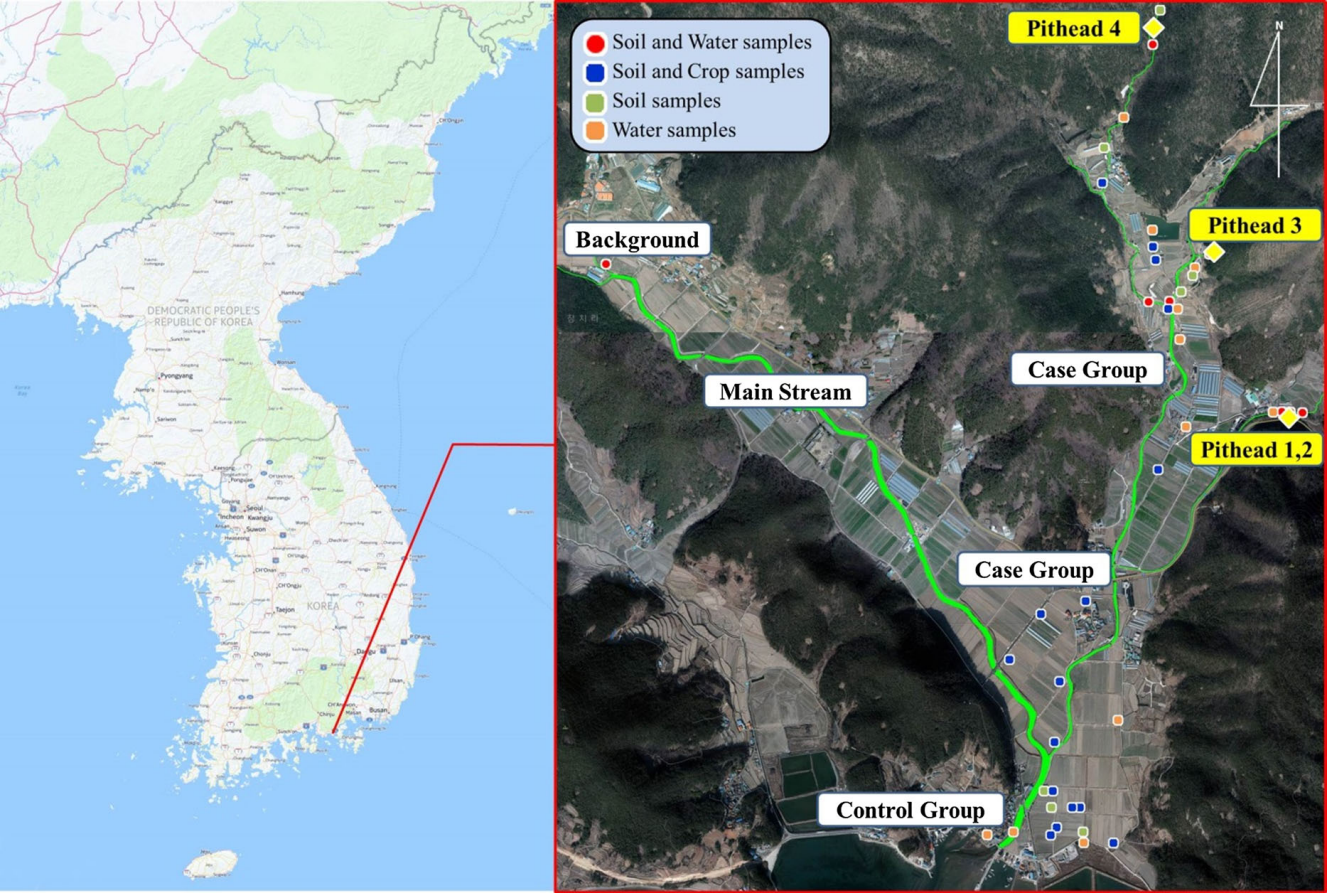
Study area and sampling site

The JH mine area was considered the case group, as the percentage of residents with blood Cd concentrations higher than the WHO reference value was found to be higher than that of other areas, as a determined by a previous study (NIER 2013a, b, c). For the control group, a village about 2 km away from the JH mine was selected; this village used different groundwater for agriculture than the case group (Fig. 1).

The survey period was from July to September, 2014, and the consent to participation, utilization of personal information, and studies on human biological materials was received from the participants. We recruited subjects who lived for a long period time in the JH mine area on a priority basis. Regarding the content and the academic and ethical aspects of this study, approval from the Institutional Review Board of the National Institute of Environment Research was obtained.

### Questionnaire

The questionnaire related to exposure to Cd was performed as one-to-one interviews. The main contents of the questionnaire included demographical/socioeconomic characteristics, occupational history, lifestyle habits, food self-sufficiency rate, and dietary habits.

### Analysis of Cd concentrations in environmental and biological samples

The concentrations of Cd in environmental and biological samples were analyzed to re-evaluate the level of Cd in blood and urine of the residents in the JH mine area and to assess their correlation with those in the abandoned metal mines.

To investigate environmental Cd contamination in the JH mine area, 35 soil samples, 19 water samples, and 55 agricultural product samples were collected. Soil samples were collected from the surroundings of the pitheads (*n* = 5), rice paddies (*n* = 25), fields (*n* = 3), and river sediments (*n* = 2); water samples were collected from the surroundings of the pitheads (*n* = 6), river (*n* = 5), reservoir (*n*  = 3), drinking water sources (*n* = 4), and a paddy field (*n* = 1). For agricultural samples, rice being cultivated (*n* = 25) and the rice grain consumed currently (*n* = 30) were used. Only one soil sample from each farmland was used in this study.

Cd concentrations in soil samples were analyzed after preprocessing them using an automatic sample degradation device; water quality was analyzed using ICP-OES (Optima 5300 DV; PerkinElmer). All soil and water samples were digested in 15 mL of trace metal-free nitric acid using the Environmental Standard Test Method in Korea (NIER 2012b). The agricultural samples such as paddy rice and rice grain, were analyzed in accordance with the Analytical Methods for Hazardous Material in Food of the Korean Food Standard Codex (KMFDS 2015) using an ICP-MS (7500 ICP-MS; Agilent Technologies).

Blood samples were collected in anticoagulant-coated vacutainer tubes; these were sufficiently agitated to prevent coagulation. Spot urine samples were collected in conical tubes. All biological samples were transported to the laboratory while being refrigerated. The samples were agitated for 1 h or longer before being analyzed. Cd concentrations in blood and urine were analyzed using the flameless method on a Graphite Furnace AAS (GF-AAS 600; PerkinElmer). The detection limits were 0.09 and 0.06 μg/L, respectively.

### Analyses of heavy metal concentration patterns in soil

An analysis of the heavy metal concentration patterns (13 heavy metals including Cd) in the soil was performed to evaluate the correlation between the soil in the JH mine area and that in the farmlands in the case and control areas.

For analyses of heavy metal concentration patterns (hierarchical clustering-nearest neighbor), we analyzed 14 soil samples (3 samples of pollution sources were collected from the JH mine, 7 samples were collected from the case area downstream of the JH mine, and 3 control and 1 background area samples were collected on the other side of JH mine) using an ICP-MS (ELAN DRC-e model; PerkinElmer) after preprocessing the soil samples in accordance with Environmental Standard Test Method in Korea (NIER 2012b). The distribution pattern of each heavy metal was analyzed using the US EPA the Fingerprint Analysis of Leachate Contaminants technique (Russell 2004; Choi et al. 2013).

### Risk assessment of the exposure factors of Cd and their contribution rates

To analyze the exposure factors of Cd in the residents in the JH mine area and their contribution rates, we used a correlation analysis of the Cd concentration between the environmental samples and biological samples, and risk assessment of each exposure pathway. The hazard quotient (HQ) used to calculate the risk level in the risk assessment for the exposure level of a pollutant is the ratio of the concentration which is expected not to cause any side effects when the subject is exposed to a chemical to the exposure level. If the HQ is 1 or less, no harmful effect on health due to exposure is expected, and if the HQ value is greater than 1, although health may be affected as the HQ value increases, it is not certain (EPA 1997b).

For risk assessment, the exposure pathway and the exposure factors were identified and exposure from the identified pathways was assessed in comparison with the tolerable daily intake. Soil ingestion, skin contact, drinking water, and consumption of agricultural products such as rice were selected as the exposure pathways that can be involved in inflow of Cd into the body, and the Cd concentrations in paddy soil, drinking water, and rice were analyzed for each selected exposure pathway. However, in the case of vegetables, as no sampling was done, the average Cd concentrations in Chinese cabbage, onion, and cucumber distributed in Korea, were used (Shim et al. 2010; Kim et al. 2009).

**Fig. 2:**
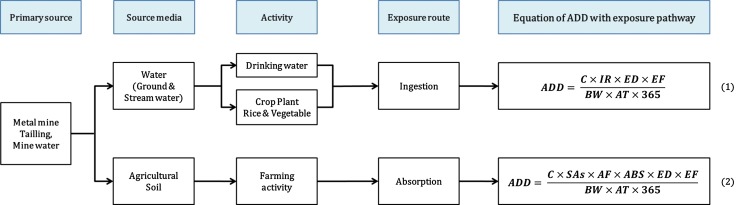
Equation of average daily dose (ADD) by exposure pathway. ADD is the average daily dose of Cd (μg/kg-day); C is concentrations of Cd in each media; IR is the ingestion rate of each media (kg); ED is exposure duration (year); EF is exposure frequency (days/year); SAs is exposure to skin surface area; AF is the soil-skin adherence factor (kg/m^2^/day); ABS is the absorption factor for skin; BW is the average body weight of the general population in Korea (kg); AT is average time (years); and 365 is the exposure days/year

The exposure coefficients used for risk level calculations are shown in Table 1, and the risk level of each exposure pathway was calculated using Eq. (1) and (2), as shown in Fig. 2.

**Table 1:** Exposure factors and parameters for risk assessment

Exposure factors	Symbol	Units	Residential	Data source
Exposure duration	ED	Years	30	EPA (1997a)
Exposure frequency	EF	Days/year	350	EPA (1997a)
Average time	AT			
Non-carcinogens	ATnc	Years	30	EPA (1997a)
Body weight	BW	kg	62.8	KMOE (2007)
Ingestion rate	IR			
Soil	IRs	kg/day	0.00002	EPA (2011)
Rice (farmer)	IRr-f	kg/day	0.296	KOSTAT (2013)
Rice (non-farmer)	IRr-nf	kg/day	0.175	KOSTAT (2013)
Napa cabbage	IRn	kg/day	0.0607	KMOHW (2013)
Onion	IRo	kg/day	0.0273	KMOHW (2013)
Drinking water	IRw	L/day	1.5	KMOE (2007)
Exposed skin surface area	SA			
Skin surface area (forearms, hands)	SAs	m^2^	1.96	EPA (1997a)
Soil-skin adherence factor	AF	kg/m^2^/day	0.0007	
Absorption factor for skin	ABSs	–	0.01	EPA (1997a)

### Statistical analysis

We used Microsoft^®^ Excel program for data management and SAS^®^ 9.4 software (SAS Institute Inc.) for statistical analysis. The correlations among categorical variables were analyzed via the Chi-square test, and for cells of which the expected observed value was less than 5, a Fisher exact test was performed. The difference between the averages of groups was verified using an independent sample *t* test and one-way analysis of variance. A two-sided *p * value < 0.05 was considered to indicate statistical significance. Urine Cd concentrations were corrected for urine creatinine concentrations before data analysis, and the Cd concentrations in biological samples were measured after log transformation.

## Result and discussion

### Status of the survey area and the participants

The JH abandoned metal mine was a gold, silver, and copper mine excavated during the 1910s and abandoned in 1980. There were 4 pitheads, and some of the mine water flowed into a river. There was no concentrator or mine tailing in the surroundings of the JH mine as the concentration work was carried out in another place after crude ore was collected from the mine, and there were no other contamination sources in the surroundings.

The case group residents were from an area in which people grew rice and used agricultural water that was contaminated with mine water; residential areas were located 1 km away from the pitheads, and the survey was performed with residents whose blood Cd concentrations exceeded the WHO reference value (WHO 1996), as measured in a previous study (NIER 2013a, b, c). The control group comprised residents living in a village located on a beach 2 km or farther away from the pitheads of the JH mine, where people cultivated rice using groundwater from the water system located opposite to the mine, thus excluding the possibility of contamination of agricultural products due to contaminated groundwater. These residents were also involved in fishing.

We recruited 37 residents into the study: 9 in the case group (average age, 71.4 years; average period of residence, 46.7 years) and 28 in the control group (average age, 65.4 years; average period of residence, 49.8 years). Among the subjects of the case group and the control group, 77.8 and 92.9 %, respectively, engaged in agriculture, and 11.1 and 14.3 %, respectively, had experience of working in a mine (Table 2).

**Table 2:** Basic characteristics of participants in this study

Factors	Case (*n* = 9)	Control (*n* = 28)	*p* value
Age (years)^a^	71.4 ± 11.7	65.4 ± 9.5	0.126^b^
Residence period (person (%))			
<40	2 (22.2)	4 (14.8)	0.717^c^
40–59	6 (66.7)	17 (63.0)	
≥60	1 (11.1)	6 (22.2)	
Average (years)	46.7 ± 16.1	49.8 ± 13.3	
Present job (person (%))			
Farmer	7 (77.8)	26 (92.9)	0.448^c^
Unemployed	2 (22.2)	2 (7.1)	
Abandoned mine (person (%))			
Working experience	1 (11.1)	4 (14.3)	1.000^c^
Working period (years)	5.0	3.0 ± 2.4	–
Smoking habit (person (%))			
Current	1 (11.1)	2 (7.1)	0.797^c^
Past	–	1 (3.6)	
Non	8 (88.9)	25 (89.3)	
Drinking habit (person (%))			
Current	–	14 (50.0)	0.014^c^
Past	1 (11.1)	4 (14.3)	
Non	8 (88.9)	10 (35.7)	

^a^Arithmetic mean ± standard deviation

^b^Independent sample *t* test

^c^Chi-square test (Fisher’s exact test)

Among the subjects in the case and control groups, 66.7 and 82.1 % used the simple water supply system as drinking water, respectively, and their self-sufficiency rate of rice and vegetables was high. In the case of fish and shellfish, the self-sufficiency rate of the control group was higher than that of the case group, which was thought to be because of the geographical location of the case group, which was near the abandoned mine, and that of the control group, which was near the sea (Table 3).

**Table 3:** Dietary questionnaire results of participants in this study

Factors	Case (*n* = 9)	Control (*n* = 28)	*p* value^a^
Drinking water (person (%))			
Tap/mineral water	3 (33.3)	5 (17.8)	0.064
Ground water	6 (66.7)	23 (82.1)	
Rice (person (%))			
All self-sufficiency	9 (100)	21 (75.0)	0.160
All purchase	–	7 (25.0)	
Vegetables (person (%))			
All self-sufficiency	7 (77.8)	26 (92.9)	0.244
All purchase	2 (22.2)	2 (7.1)	
Fish (person (%))			
All self-sufficiency	–	11 (39.3)	0.077
Half self-sufficiency	2 (22.2)	3 (10.7)	
All purchase	7 (77.8)	14 (50.0)	
Shellfish (person (%))			
All self-sufficiency	2 (22.2)	23 (82.1)	0.002
All purchase	7 (77.8)	5 (17.9)	

^a^Chi-square test (Fisher’s exact test)

### Cd contamination of environmental media

In the case of environmental samples in the case group, although the average Cd concentrations of the paddy soil (1.29 mg/kg) and the rice grain (0.14 mg/kg) consumed by the residents in the case area did not exceed the worrisome level of soil contamination in Korea (KMOE, 4 mg/kg) and the standard for Cd level in agricultural product (KMFDS, 0.2 mg/kg), they were significantly higher than the control group (0.91 and 0.07 mg/kg, respectively). In addition, the levels were higher than Korean paddy soil (0.85 mg/kg) and general rice grain (0.021 mg/kg) (NIER 2013b; Kim et al. 2009). The concentrations of Cd in the water samples were all below the detection limits (Table 4).

**Table 4:** Cd concentrations in environmental samples

Factors	Group	*N*	AM^a^	Min–Max^b^	*p *value^c^
Paddy soil (mg/kg)	Case	8	1.29 ± 0.39	0.93–2.17	0.029
	Control	17	0.91 ± 0.10	0.66–1.02	
Rice grain (mg/kg)	Case	8	0.14 ± 0.03	0.10–0.18	0.000
	Control	22	0.07 ± 0.03	0.03–0.13	

^a^Arithmetic mean ± standard deviation

^b^Minimum–maximum

^c^Independent sample *t* test

According to the Soil Environment Conservation Act in Korea, there are two types of environmental guidelines determined by the Ordinance of KMOE; the worrisome level and the countermeasure standard of soil contamination (KMOE 2016). The worrisome level is likely to obstruct the health and properties of persons or rearing of animals and plants. The countermeasure standard is likely to obstruct the health and properties of persons or rearing of animals and plant, and would accordingly necessitate countermeasures.

The Cd concentrations in the soil of the JH mine area were higher than the results of the survey of heavy metal contamination of paddy soil in Korea (NIER 2013b). Only one field sample exceeded the worrisome level of soil contamination.

The Cd concentration in the rice grain consumed by the case group was 0.14 mg/kg and that of the control group was 0.07 mg/kg. These concentrations were higher than the Cd average concentration (0.021 mg/kg) in rice grain in Korea (Kim et al. 2009), though they did not exceed 0.2 mg/kg (KMFDS 2015).

As only one soil sample from each farmland was used in this study, the results may not be generalizable to the entire JH mine area, as the crop had not been harvested at the time of the survey and the samples of the rice consumed by the residents were those that the participants brought to the survey site. However, it could be confirmed that the heavy metal contaminations in the environmental factors of the case group, which was an area in the vicinity of the JH mine, had higher values than those of the control group.

### Cd concentration in biological samples

The Cd concentrations in the blood and urine of the participants are listed in Table 5. The geometric mean of the Cd concentrations in the blood and urine samples of the case group residents were 5.33 μg/L and 6.19 μg/g-creatinine (μg/g-cr), respectively, which were significantly higher than those of the control group residents (1.63 μg/L and 1.16 μg/g-cr, respectively). The blood Cd concentrations exceeded the WHO reference value of 5 μg/L in five subjects in the case group (WHO 1996), and of these five, the urine Cd concentrations of four subjects exceeded 5 μg/g-cr, the Biological Exposure Indices of the American Conference of Governmental Industrial Hygienist (ACGIH 2012).

**Table 5:** Cd concentrations in biological samples of participants

Metals^a^	Group	*N*	AM ± SD^b^	G.M (95 % C.I)^c^	Min–Max^d^	*p* value^e^
B-Cd (μg/L)	Case	9	6.05 ± 3.13	5.33 (3.63, 7.45)	2.29–11.66	0.000
	Control	28	1.99 ± 1.12	1.63 (1.23, 2.08)	0.20–4.04	
U-Cd (μg/L)	Case	9	7.26 ± 4.32	5.31 (2.57, 9.80)	0.67–11.93	0.003
	Control	28	2.95 ± 3.51	1.02 (0.45, 2.04)	ND^f^–15.46	
U-Cd (μg/g-cr)	Case	9	7.43 ± 4.59	6.19 (3.76, 9.13)	1.44–17.67	0.000
	Control	26	2.53 ± 2.50	1.16 (0.61, 2.05)	0.05–7.73	

^a^ *B*-*Cd* blood Cd concentration, *U*-*Cd* urine Cd Concentration

^b^Arithmetic mean ± standard deviation

^c^Geometric mean (95 % Confidence Interval)

^d^Minimum–maximum

^e^Independent sample t test

^f^Not detected

The health problems of the residents in abandoned metal mine areas were mostly attributable to exposure to Cd, as seen in the cases of the Gahak, Sucheol, and Samsan Jeil mines. The urine Cd concentrations of the residents living in the Gahak abandoned mine area were higher than those of residents living in the control area, and some residents showed signs of renal dysfunction (Park et al. 1998). In addition, the urine Cd concentrations of the residents in the Sucheol abandoned mine and Samsan Jeil mine areas were also significantly higher than those of residents in the control areas (Chung et al. 2005; Kim et al. 2008).

The blood Cd concentration of the Korean general population aged ≥20 years was 0.98 μg/L and the urine Cd concentration was 0.66 μg/g-cr (KMOWH 2013; NIER 2013c). The blood Cd concentrations of the 5682 residents living near the 38 abandoned metal mines in Korea were found to be 1.60 μg/L, and those of the residents living within a 2 km radius of the abandoned mine were found to be 1.87 μg/L, which are higher than those of the residents living outside of the 2 km radius (1.31 μg/L) (Park et al. 2014). The blood Cd concentration of the US general population aged ≥20 years was 0.36–0.47 μg/L (CDC CDC 2009), while that of the Canadian general population aged ≥20 years was 0.35–0.49 μg/L. The urine Cd concentration was 0.31–0.70 μg/g-cr (Health Canada 2010). In Germany, the blood Cd concentration among smokers aged ≥18 years was 1.06 μg/L and that among non-smokers was 0.28 μg/L (Becker et al. 2002). The blood Cd concentrations of the case and control subjects in this study were 5.33 and 1.63 μg/L, respectively, and the urine Cd concentrations were 6.19 and 1.16 μg/g-cr, respectively; these were higher than those of the general population of Korea, USA, Canada, and Germany. Furthermore, the levels were higher than those of the residents in other abandoned mine areas.

### Analyses of the heavy metal concentration pattern in soil

Regarding the results of analyses of heavy metal concentration patterns in soil, the levels of Cd in the paddy soil of the case group were higher than those of the control group by 4.6 times on average. In addition, levels of lead, zinc, and manganese in the case area soil were higher than those in the control area soil by 2.1, 2.0, and 1.6 times on average, respectively (data not shown).

**Fig. 3:**
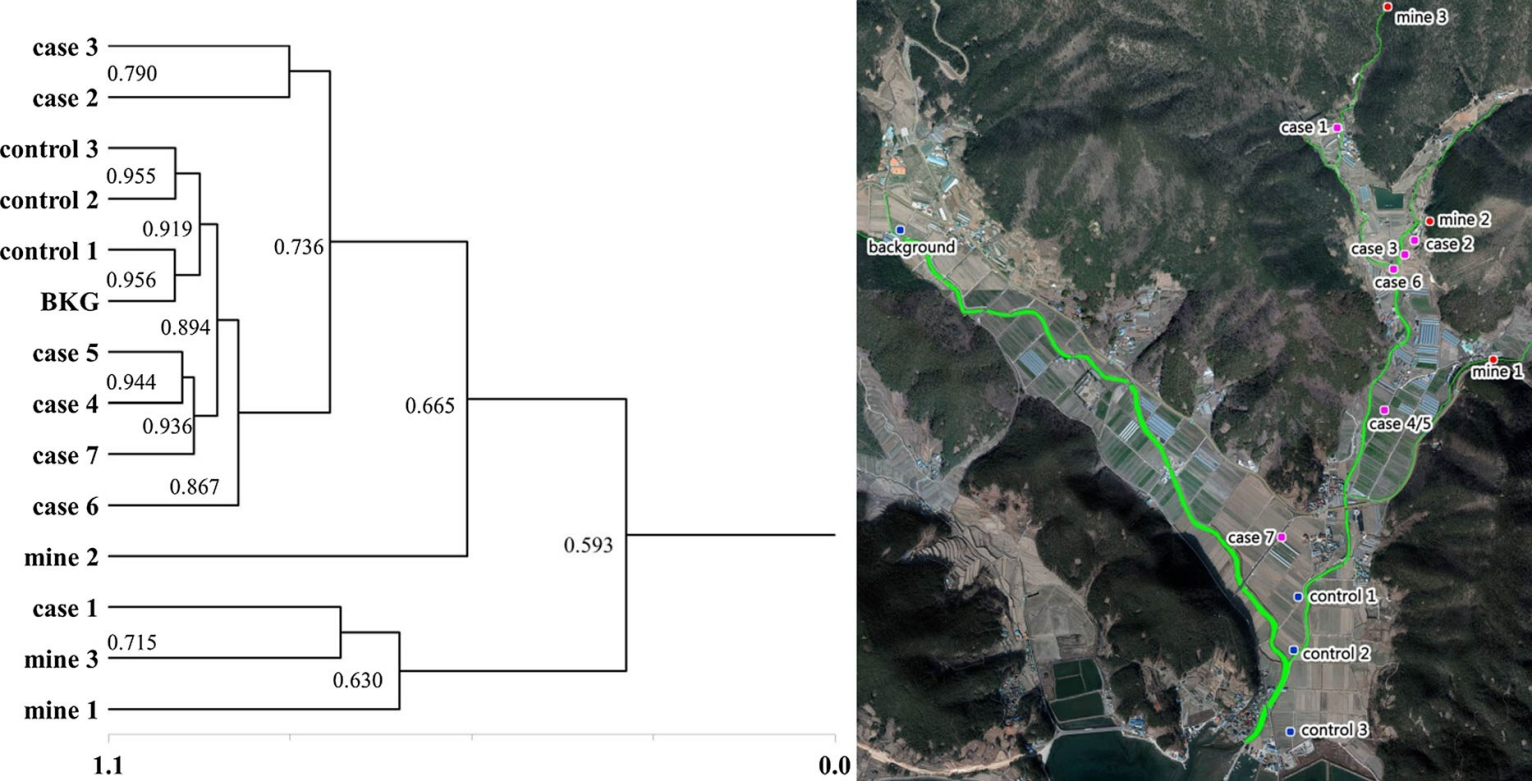
Dendrogram of the data set of relative ratios for heavy metal concentration in soil samples

Although all the samples were thought to have been affected by the JH mine to some extent (similarity > 0.50), the pattern analysis of heavy metal concentrations in each sample, showed greater correlations between the pithead soil of the JH mine, which was the pollution source, and the paddy soil of the case group (similarity < 0.90); the paddy soil of the control group was shown to have higher correlations with that of the background area (similarity > 0.90) compared to that of the case group (Table 6; Fig. 3). Thus, it can be inferred that the paddies of the case group were affected more by the JH mine than those of the control group.

**Table 6:** Results of similarity matrix of soil samples

	Mine 1	Mine 2	Mine 3	BKG	Case 1	Case 2	Case 3	Case 4	Case 5	Case 6	Case 7	Control 1	Control 2	Control 3
Mine 1		0.593	0.630	0.587	0.667	0.645	0.602	0.646	0.623	0.655	0.653	0.600	0.603	0.598
Mine 2	0.593		0.303	0.611	0.444	0.532	0.665	0.637	0.636	0.668	0.626	0.627	0.627	0.642
Mine 3	0.630	0.303		0.395	0.715	0.479	0.358	0.450	0.412	0.434	0.467	0.401	0.412	0.421
BKG^a^	0.587	0.611	0.395		0.655	0.731	0.755	0.917	0.934	0.891	0.894	0.956	0.919	0.930
Case 1	0.667	0.444	0.715	0.655		0.737	0.596	0.704	0.663	0.684	0.732	0.662	0.672	0.682
Case 2	0.645	0.532	0.479	0.731	0.737		0.790	0.790	0.775	0.736	0.812	0.758	0.789	0.763
Case 3	0.6302	0.665	0.358	0.755	0.596	0.790		0.799	0.809	0.774	0.805	0.785	0.796	0.786
Case 4	0.646	0.637	0.450	0.917	0.704	0.790	0.799		0.944	0.902	0.936	0.907	0.925	0.927
Case 5	0.623	0.636	0.412	0.937	0.663	0.775	0.809	0.944		0.897	0.928	0.931	0.936	0.931
Case 6	0.655	0.668	0.434	0.891	0.684	0.736	0.774	0.902	0.897		0.867	0.874	0.863	0.867
Case 7	0.653	0.626	0.467	0.894	0.732	0.812	0.805	0.936	0.928	0.867		0.917	0.911	0.915
Control 1	0.600	0.627	0.401	0.956	0.662	0.758	0.785	0.907	0.931	0.874	0.917		0.931	0.937
Control 2	0.603	0.627	0.412	0.919	0.672	0.789	0.796	0.925	0.936	0.863	0.911	0.931		0.955
Control 3	0.598	0.642	0.421	0.930	0.682	0.763	0.786	0.927	0.931	0.867	0.915	0.937	0.955	

^a^Background area

Based on the results of the analysis of the distribution patterns of heavy metal concentrations in soil, the concentrations of Cd and other heavy metals in the soil in the case area were thought to have been affected by the JH abandoned metal mine.

### Risk assessment of exposure factors of Cd and evaluation of the contribution rate for each factor

An analysis of the correlation between Cd concentrations in the environmental media and biological samples showed a significant correlation between Cd concentrations in the paddy soil and rice grains (*p* = 0.049), rice grain and urine Cd (*p* = 0.039) and blood Cd (*p* < 0.01), and urine Cd and blood Cd (*p* < 0.01).

A comparison of the Cd concentrations in the environmental media revealed that Cd concentrations in the paddy soil of the case group and the rice consumed by the residents were 1.29 and 0.14 mg/kg, respectively, which were significantly higher than 0.89 and 0.06 mg/kg in the control group (Table 7).

**Table 7:** Cd concentrations in environmental samples used for risk assessment

Metals	Group	Paddy soil	Ground water	Rice grain	Napa cabbage	Onion	Cucumber
Cd (mg/kg)	Case	1.29	ND^b^	0.14	0.007	0.006	0.002
	Control	0.89	ND^b^	0.06	0.007	0.006	0.002
	*p* value^a^	0.026	–	0.000	–	–	–

^a^Independent sample *t* test

^b^Not detected

Non-carcinogenic risk assessment for the major pollution sources, taking into account the exposure path, revealed that in case of Cd, the HQs of the case group and the control group due to consumption of rice were 1.25 and 0.6, respectively (Table 8). The HQ of Cd exposure due to consumption of rice was 97 % or more of the total HQ in both groups and that of the case group was 2 times higher than that of the control group, showing a trend similar to that of the occurrence rate of subjects whose blood Cd was higher than the reference value. The correlation analysis for Cd concentrations in the biological and environmental samples and the risk assessment for the exposure factors showed that rice grain was the major exposure factor.

**Table 8:** Results of risk assessment according to exposure source and pathway

Metals	Cd	
Group	Case	Control
HQ		
Soil ingestion	7.86E−04	5.46E−04
Soil dermal contact	5.39E−04	3.75E−04
Water ingestion	0.00E+00	0.00E+00
Rice ingestion	1.25E+00	6.00E−01
Napa cabbage ingestion	1.30E−02	1.30E−02
Onion ingestion	5.00E−03	5.00E−03
Cucumber ingestion	9.77E−04	9.77E−04
Total HQ	1.27E+00	6.20E−01

The HQs in previous studies on the residents of domestic abandoned metal mines were lower than those of this study, with values of 0.001–0.8, about 99 % of the daily intake Cd dose resulted from consumption of agricultural products, which is an observation similar to that in this study (Yang et al. 2015). In addition, the HQ of a study on gold and silver mines similar to the JH mine was 4.0, indicating that the main cause was consumption of contaminated drinking water (Lee et al. 2005).

However, the limitation of this study is that the risk assessment may be unreliable as the number of the environmental and agricultural product samples used for the non-carcinogenic risk assessment was small and the average Cd concentrations of some agricultural products consumed in Korea, not of the relevant area, were used. The effect may have been overestimated as total Cd exposure through soil, water, agricultural products, and so forth was assumed to result in absorption.

## Conclusion

In this study, the correlation between the factors of exposure to Cd among abandoned mine area residents and the abandoned mine was evaluated. The blood and urine Cd concentrations of the residents of the JH mine area were significantly higher than those of the residents of the control area, and the Cd contamination in environmental media of the JH mine area was also high in comparison to the control area. In the risk assessment of the main exposure factors such, as soil, drinking water, rice grain, among others, by exposure path, rice grain was determined to be the main exposure factor, as consumption of Cd-contaminated rice accounted for 97 % of the total HQ. Furthermore, an analysis of the distribution pattern of the Cd concentrations in soil revealed that the blood Cd concentrations in the residents of the JH mine area were affected by the mine to some extent, as the Cd levels in farmland soil were found to have been affected by the mine.

The significance of this study is that the correlation between exposure to Cd and abandoned metal mines has been identified through the analysis of factors of exposure to Cd among the abandoned mine area residents. However, it can be concluded that the main cause for the exposure of the residents in the JH abandoned metal mine area to Cd was regular consumption of rice cultivated in Cd-exposed soil.
